# Supplementary material for: Transcriptomic Insights into Mechanisms of Early Seed Maturation in the Garden Pea (Pisum sativum L.)
Source: Cells. 2020 Mar 23;9(3):779. doi: 10.3390/cells9030779 (PMC7140803; doi:10.3390/cells9030779)
Supplement: Supplementary file 1 [file cells-09-00779-s001.zip › Supplementary materials/FileS4.zip!/FileS3/TFs - Results/Supporting Maps&Profiles/Entropy Profiles.pdf]

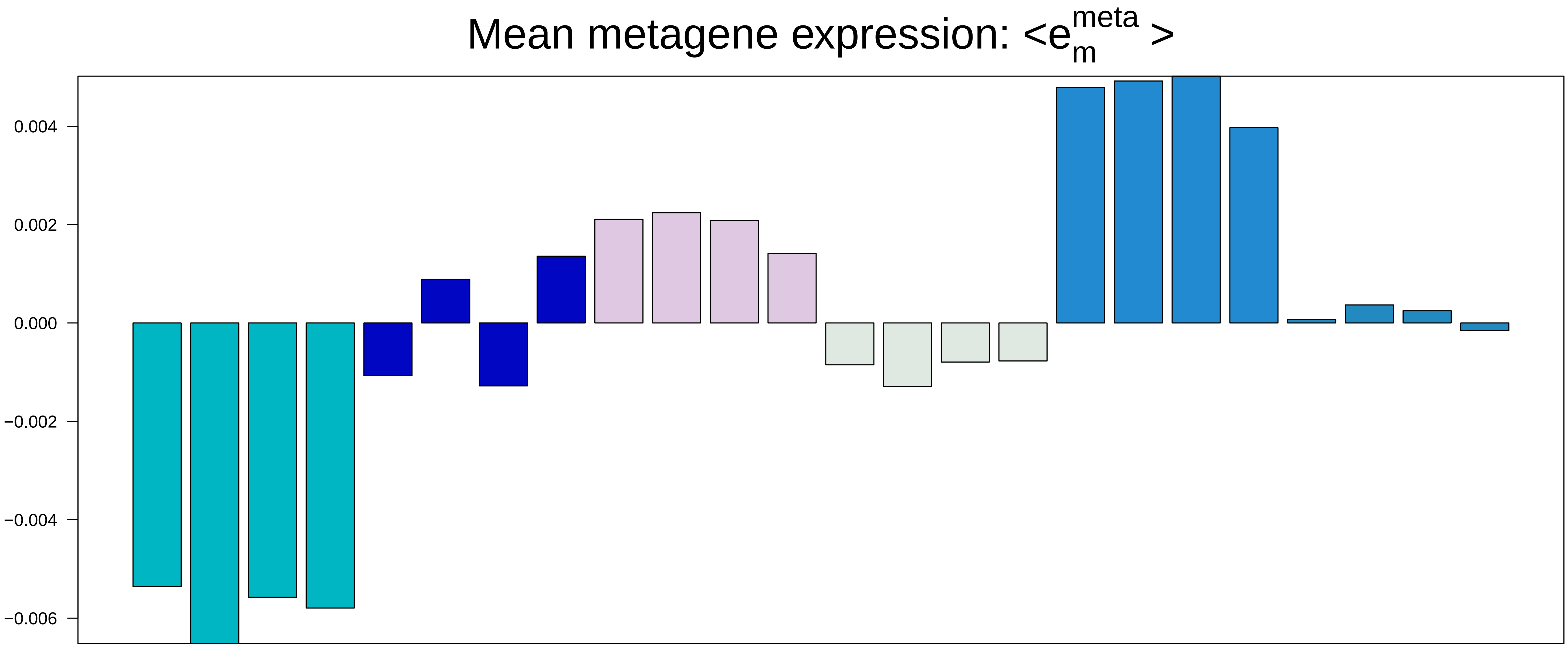

Mean metagene expression: <e_m^meta>
0.004
0.002
0.000
−0.002
−0.004
−0.006

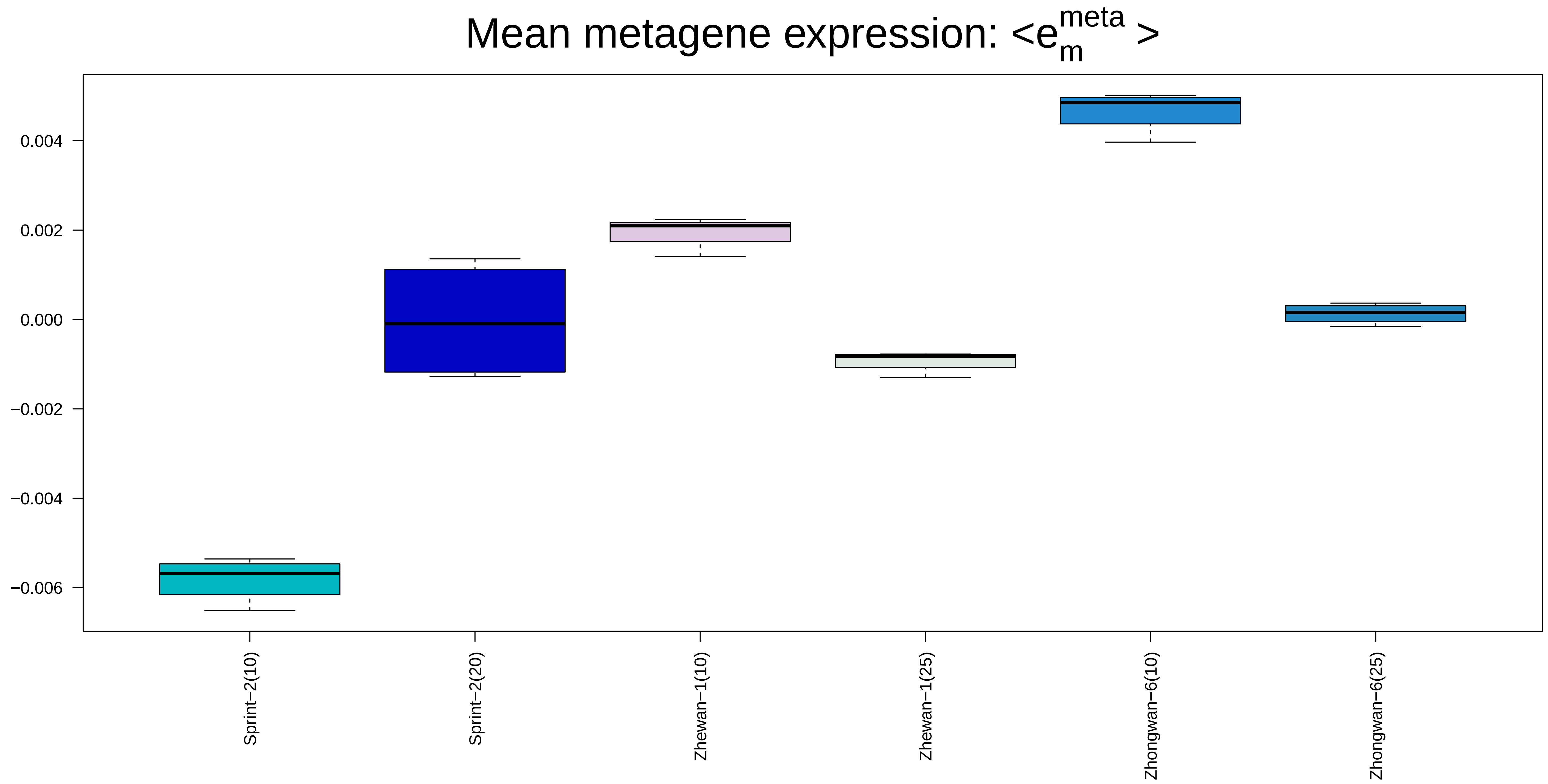

Mean metagene expression: $<e_m^{meta}>$
0.004
0.002
0.000
−0.002
−0.004
−0.006
Sprint−2(10)
Sprint−2(20)
Zhewan−1(10)
Zhewan−1(25)
Zhongwan−6(10)
Zhongwan−6(25)

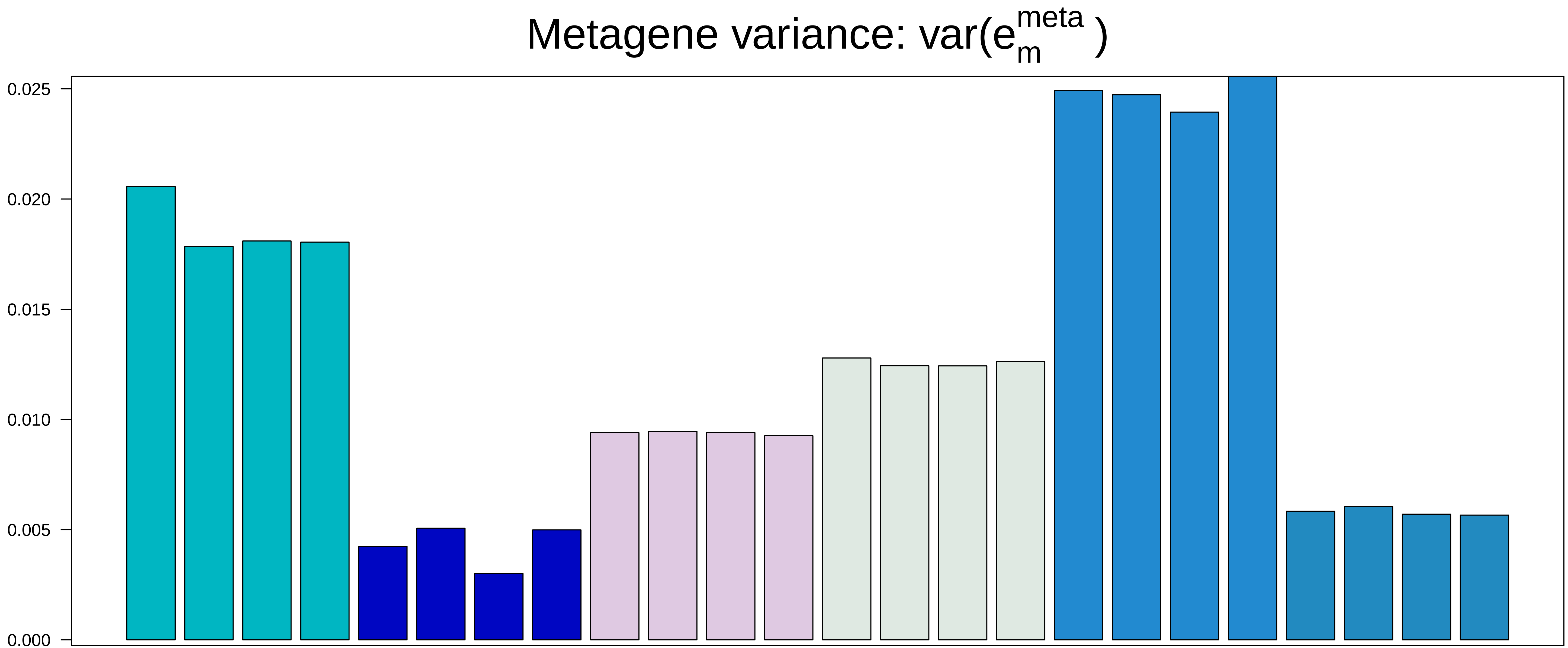

Metagene variance: var(e_m^{meta})
0.025
0.020
0.015
0.010
0.005
0.000

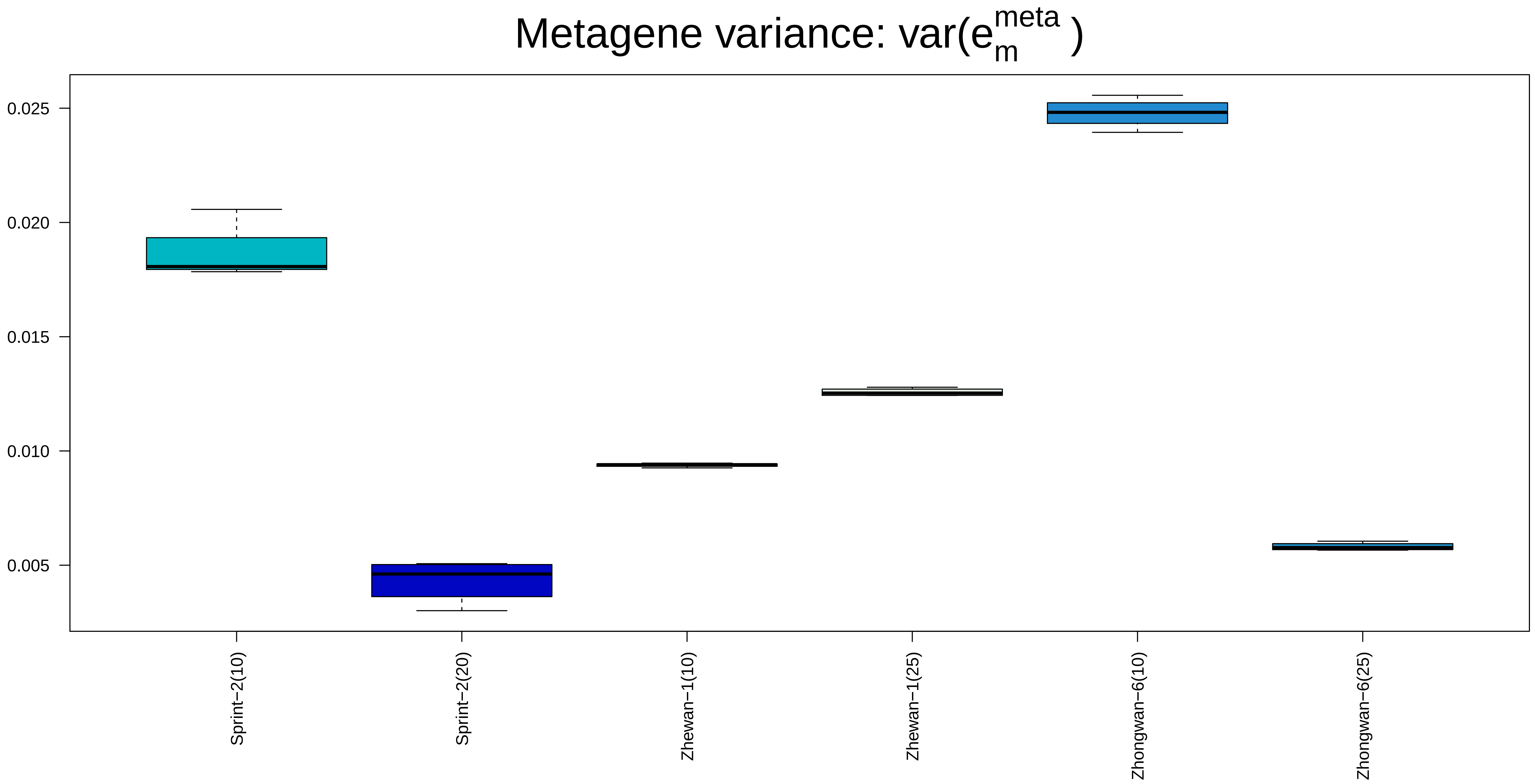

Metagene variance: var(e_m^{meta})
0.025
0.020
0.015
0.010
0.005
Sprint−2(10)
Sprint−2(20)
Zhewan−1(10)
Zhewan−1(25)
Zhongwan−6(10)
Zhongwan−6(25)

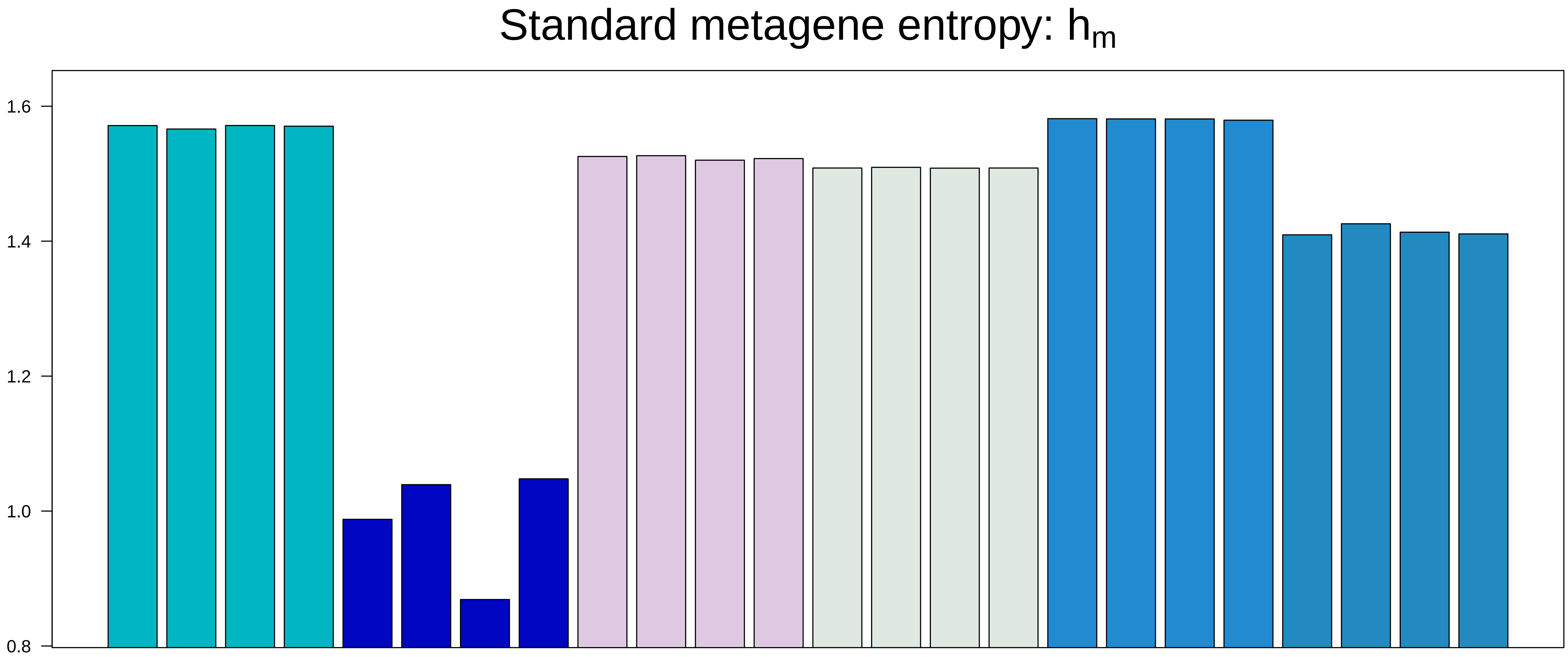
Standard metagene entropy: h_m
1.6
1.4
1.2
1.0
0.8

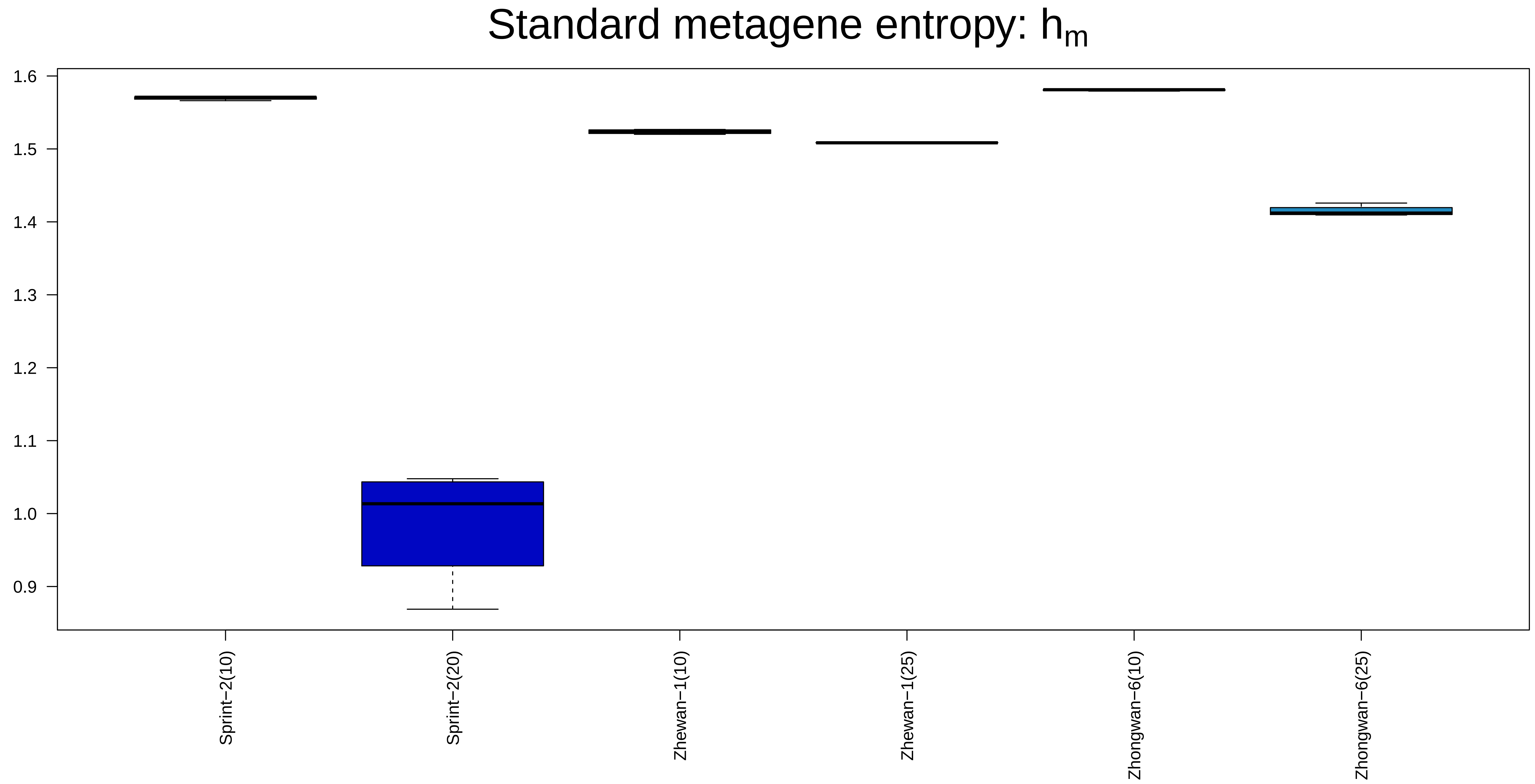

Standard metagene entropy: h_m
1.6
1.5
1.4
1.3
1.2
1.1
1.0
0.9
Sprint−2(10)
Sprint−2(20)
Zhewan−1(10)
Zhewan−1(25)
Zhongwan−6(10)
Zhongwan−6(25)

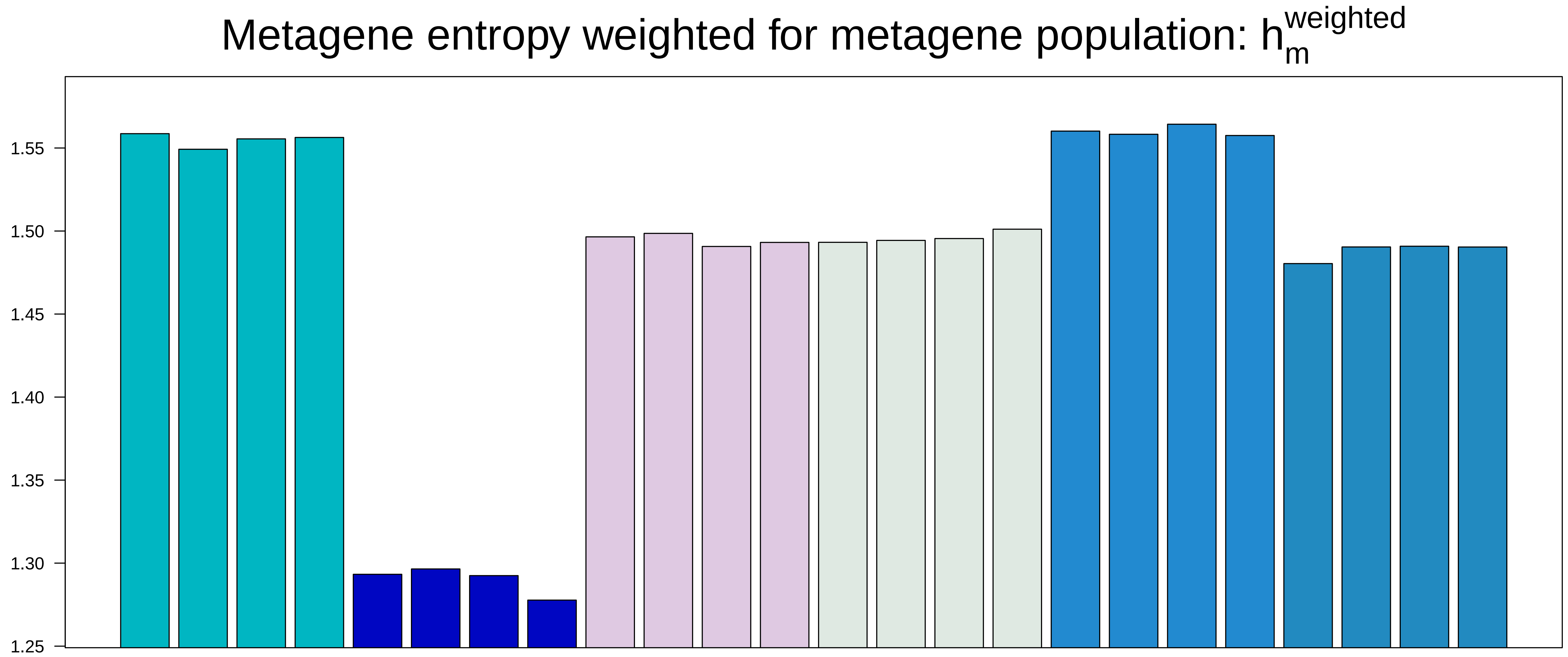
Metagene entropy weighted for metagene population: $h_m^{weighted}$
1.55
1.50
1.45
1.40
1.35
1.30
1.25

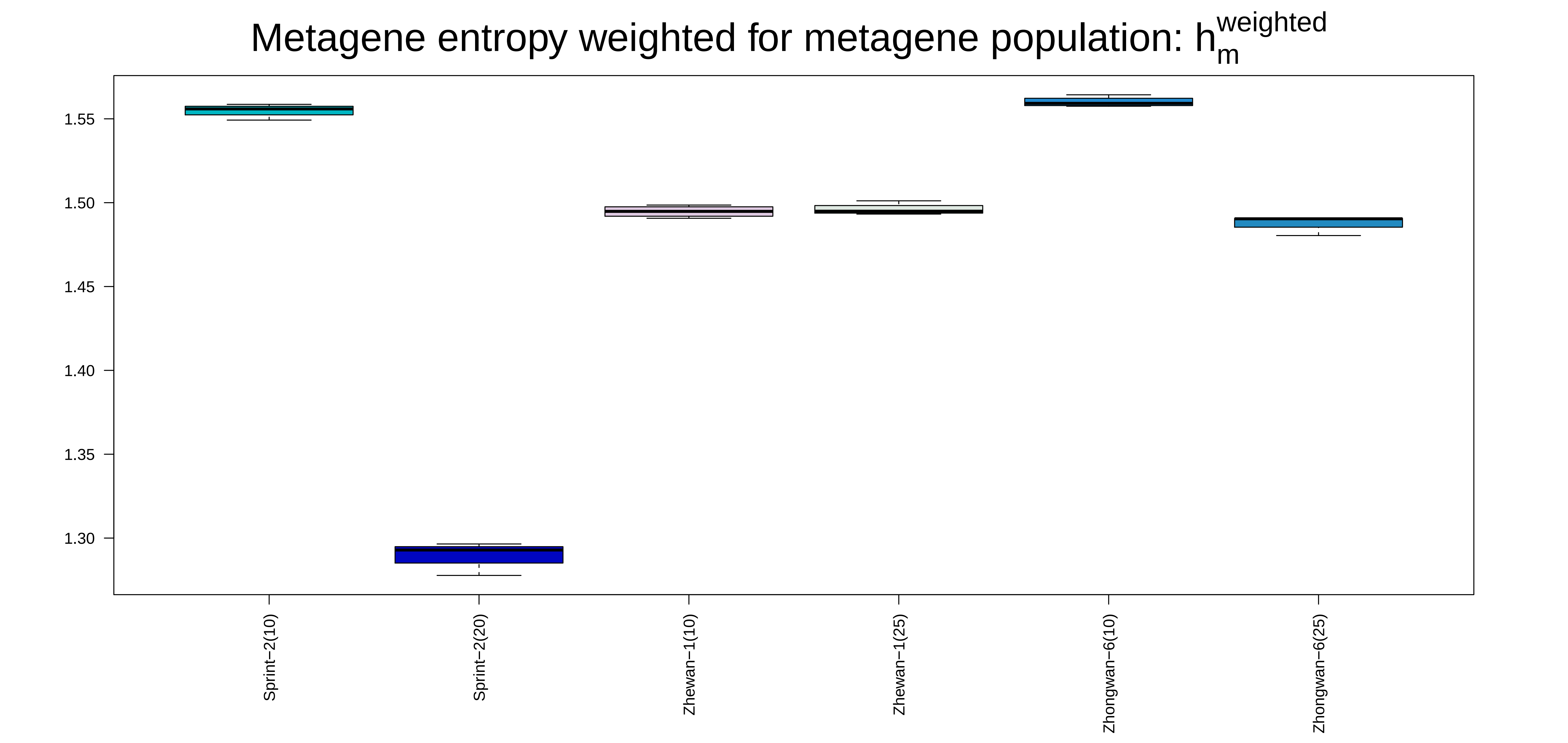
Metagene entropy weighted for metagene population: $h_m^{weighted}$
1.55
1.50
1.45
1.40
1.35
1.30
Sprint−2(10)
Sprint−2(20)
Zhewan−1(10)
Zhewan−1(25)
Zhongwan−6(10)
Zhongwan−6(25)

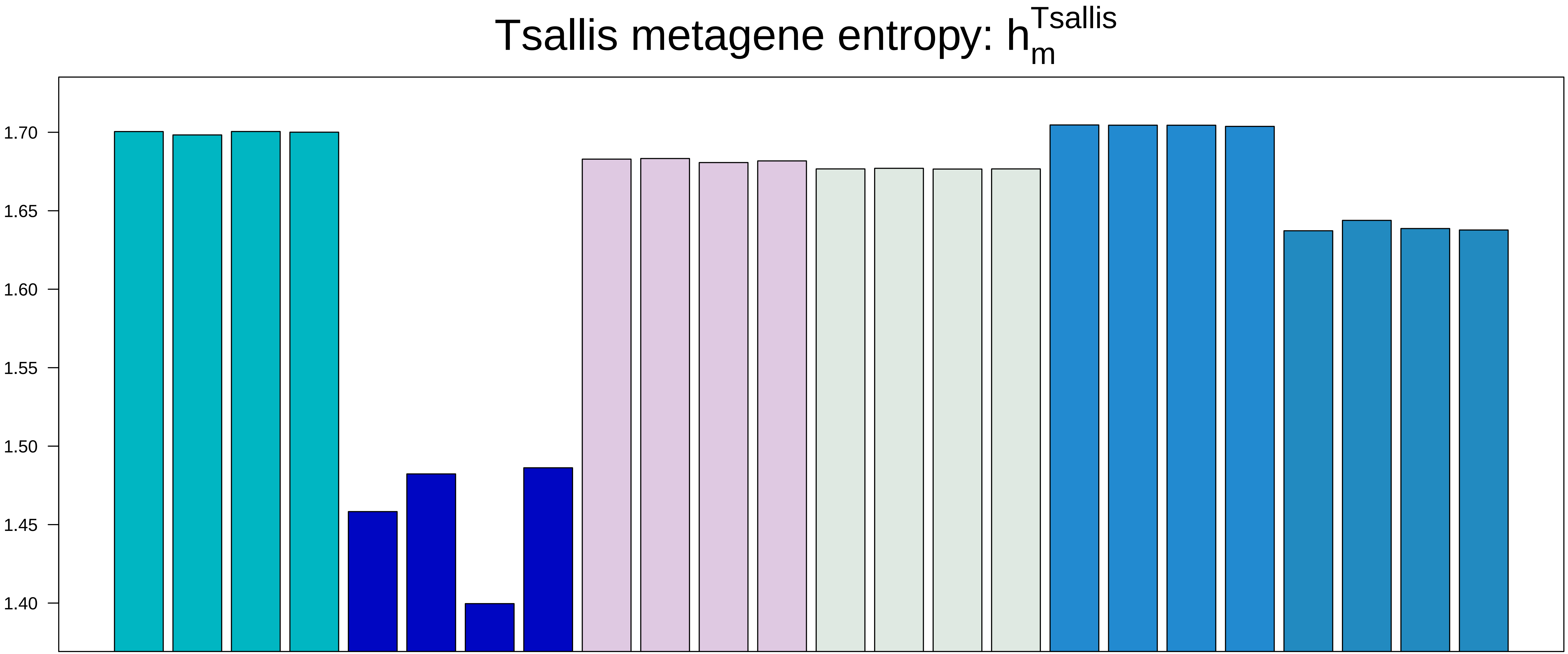

Tsallis metagene entropy: $h_m^{Tsallis}$
1.70
1.65
1.60
1.55
1.50
1.45
1.40

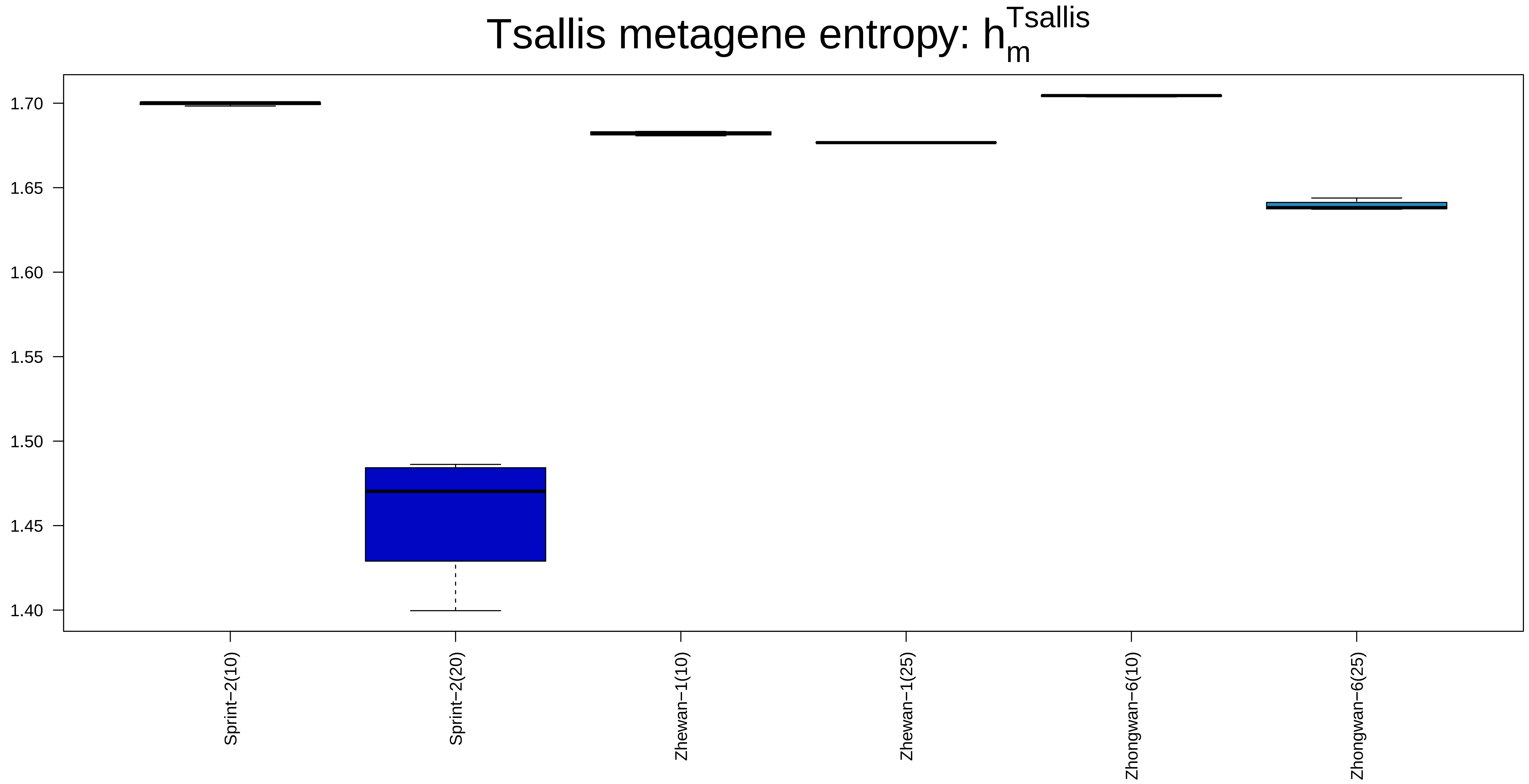

Tsallis metagene entropy: $h_m^{Tsallis}$
1.70
1.65
1.60
1.55
1.50
1.45
1.40
Sprint−2(10)
Sprint−2(20)
Zhewan−1(10)
Zhewan−1(25)
Zhongwan−6(10)
Zhongwan−6(25)
